# HHLA2 as an emerging immune checkpoint in lung cancer: linking EGFR signaling, macrophage polarization, and CD8^+^ T-cell metabolic suppression

**DOI:** 10.3389/fimmu.2026.1815639

**Published:** 2026-05-25

**Authors:** Juan Wang, Kang Wang, Zhenhong Hu, Xueting Hu

**Affiliations:** 1 Department of Respiratory and Critical Care Medicine, General Hospital of Central Theater Command, PLA, Wuhan, China; 2 Department of Respiratory and Critical Care Medicine, The First Affiliated Hospital of Army Medical University, Chongqing, China

**Keywords:** EGFR-mutant lung cancer, HHLA2, KIR3DL3, T-cell metabolism, tumor-associated macrophages

## Abstract

Immune checkpoint blockade has revolutionized the treatment of lung cancer; however, therapeutic responses remain heterogeneous, particularly in patients harboring activating epidermal growth factor receptor (EGFR) mutations. Emerging evidence identifies human endogenous retrovirus-H long terminal repeat-associating protein 2 (HHLA2), a novel B7 family member, as a genotype-associated immune checkpoint enriched in EGFR-mutant lung cancer. Beyond its classical role in T-cell inhibition, HHLA2 appears to integrate tumor-intrinsic oncogenic signaling with immune microenvironment remodeling. Recent studies demonstrate that HHLA2 promotes tumor progression by enhancing EGFR/MAPK/ERK signaling, thereby supporting proliferation, invasion, and epithelial–mesenchymal transition. In parallel, HHLA2 reshapes the tumor microenvironment through interleukin-10–dependent macrophage M2 polarization, contributing to an immunosuppressive niche. Notably, the HHLA2–KIR3DL3 axis directly suppresses CD8^+^ T-cell glutamine metabolism, introducing a metabolic checkpoint mechanism that limits cytotoxic function and facilitates immune evasion. Clinically, lower HHLA2 expression has been associated with improved response to neoadjuvant immunotherapy and increased tissue-resident memory T-cell infiltration, suggesting predictive biomarker potential. Collectively, HHLA2 represents a multifunctional immune checkpoint that links oncogenic EGFR signaling, macrophage polarization, and metabolic suppression of CD8^+^ T cells. Targeting the HHLA2–KIR3DL3 axis, particularly in combination with EGFR tyrosine kinase inhibitors, may provide a rational precision immunotherapy strategy for EGFR-mutant lung cancer. Further mechanistic and translational studies are warranted to fully define its therapeutic value.

## Highlights

- HHLA2 is enriched in EGFR-mutant lung cancer and functions as a genotype-associated immune checkpoint.
- HHLA2 promotes tumor progression by enhancing EGFR signaling and driving IL-10–dependent macrophage M2 polarization.
- The HHLA2–KIR3DL3 axis suppresses CD8^+^ T-cell glutamine metabolism, representing a metabolic immune checkpoint with therapeutic potential.

## 1 Introduction

Immune checkpoint blockade has transformed the therapeutic landscape of lung cancer over the past decade (1–3). Antibodies targeting the PD-1/PD-L1 axis have significantly improved survival in subsets of patients with advanced non-small cell lung cancer (NSCLC), establishing immunotherapy as a cornerstone of modern treatment strategies (4, 5). However, clinical benefit remains heterogeneous. A particularly challenging subgroup comprises patients harboring activating mutations in the epidermal growth factor receptor (EGFR). Despite the success of EGFR tyrosine kinase inhibitors (TKIs), EGFR-mutant tumors generally exhibit limited responsiveness to immune checkpoint inhibitors (ICIs), even in the presence of PD-L1 expression (6, 7). The mechanisms underlying this immune resistance are incompletely understood but are believed to involve a unique tumor immune microenvironment characterized by reduced cytotoxic T-cell infiltration, impaired antigen presentation, and enrichment of immunosuppressive cell populations. These observations highlight the need to identify genotype-associated immune suppressive pathways that operate specifically in EGFR-driven lung cancer.

Human endogenous retrovirus-H long terminal repeat-associating protein 2 (HHLA2) has recently emerged as a novel immune checkpoint molecule belonging to the B7 family (8, 9). Unlike classical B7 ligands, HHLA2 is evolutionarily restricted to higher primates and signals through distinct receptors, including the inhibitory receptor KIR3DL3 and the co-stimulatory receptor TMIGD2 (also known as CD28H) (9, 10). Increasing evidence demonstrates that HHLA2 is widely expressed in human lung cancers and is significantly associated with EGFR mutational status, suggesting a potential link between oncogenic signaling and immune regulation (11–13). Beyond its canonical role in modulating T-cell responses, recent studies indicate that HHLA2 may also exert tumor-intrinsic effects, influencing EGFR/MAPK/ERK signaling pathways, regulating cytokine production such as interleukin-10, and reshaping the tumor microenvironment through macrophage polarization (14). Furthermore, the HHLA2–KIR3DL3 axis has been implicated in suppressing CD8^+^ T-cell glutamine metabolism, thereby introducing a metabolic dimension to immune escape (15).

This review summarizes recent advances highlighting HHLA2 as a genotype-associated immune checkpoint that integrates oncogenic EGFR signaling, tumor-associated macrophage polarization, and CD8^+^ T-cell metabolic suppression in lung cancer.

## 2 HHLA2 expression landscape in lung cancer

Accumulating evidence indicates that HHLA2 is broadly expressed across lung cancer subtypes, with distinct associations to molecular and clinical characteristics. In a landmark study published in Clinical Cancer Research (2017), Cheng et al. systematically evaluated HHLA2 expression in human lung cancer tissues using immunohistochemistry and demonstrated that HHLA2 is widely expressed in non-small cell lung cancer (NSCLC), while largely absent or weakly expressed in normal lung epithelium (14). Notably, approximately two-thirds of NSCLC samples exhibited positive HHLA2 staining. Importantly, HHLA2 expression was significantly enriched in tumors harboring activating EGFR mutations. Multivariate analyses confirmed EGFR mutational status as an independent factor associated with high HHLA2 expression, suggesting a genotype-specific immune regulatory landscape in EGFR-driven lung adenocarcinoma. These findings established the first clinical link between HHLA2 and oncogenic EGFR signaling, positioning HHLA2 as a potential immune checkpoint preferentially active in molecularly defined subgroups of NSCLC.

Beyond NSCLC, emerging data indicate that HHLA2 is also expressed in small cell lung cancer (SCLC), a biologically aggressive subtype characterized by rapid progression and early metastasis. A recent study published in Technol Cancer Res Treat (2024) analyzed HHLA2 expression in SCLC tumor specimens and reported that positive HHLA2 expression correlated with larger tumor size, advanced clinical stage, and inferior overall survival (16). Patients with HHLA2-positive tumors exhibited significantly shorter two-year survival compared with those lacking HHLA2 expression. Moreover, HHLA2 expression was associated with elevated serum neuron-specific enolase (NSE) levels. NSE is a commonly used serum biomarker for small cell lung cancer that reflects neuroendocrine differentiation and is often associated with tumor burden, disease progression, and treatment monitoring (17–19). This association further supports the link between HHLA2 expression and aggressive tumor biology in SCLC. Although mechanistic studies in SCLC remain limited, these clinical observations suggest that HHLA2 may serve as a prognostic biomarker across histologic subtypes of lung cancer.

At a broader level, a meta-analysis published in Medicine (2021), focusing on solid tumors within the Chinese population, further supported the prognostic relevance of HHLA2 (20). While the direction and magnitude of its prognostic impact appeared tumor-type dependent, elevated HHLA2 expression was generally associated with unfavorable outcomes in several malignancies, reinforcing its potential as a clinically meaningful immune checkpoint molecule. Collectively, current evidence supports several key concepts: (1) HHLA2 is frequently expressed in NSCLC and SCLC; (2) its expression is particularly enriched in EGFR-mutant NSCLC; and (3) higher HHLA2 levels are associated with advanced disease features and poorer prognosis. These findings underscore HHLA2 not only as an immune regulatory molecule but also as a potential biomarker for molecular stratification and risk assessment in lung cancer.

## 3 HHLA2 and tumor-intrinsic oncogenic signaling

Although HHLA2 is primarily recognized as an immune checkpoint ligand, emerging evidence suggests that it may also exert tumor-intrinsic functions that extend beyond immune modulation (8, 21, 22). This concept is particularly intriguing in the context of lung cancer, where oncogenic signaling and immune regulation are often tightly intertwined. A pivotal study published in Cancer Medicine (2021) provided the first functional evidence that HHLA2 may actively participate in regulating oncogenic signaling pathways within tumor cells themselves (13). In this study, genetic silencing of HHLA2 in NSCLC cell lines resulted in a marked reduction in malignant phenotypes, including decreased proliferation, migration, and invasion. Mechanistically, HHLA2 knockdown led to significant downregulation of phosphorylated EGFR, MEK, and ERK, while total protein levels remained largely unchanged. These findings suggest that HHLA2 does not regulate receptor abundance per se but may influence activation status within the EGFR/MAPK/ERK signaling cascade. Given the central role of EGFR signaling in lung adenocarcinoma—particularly in EGFR-mutant tumors—this observation provides a compelling link between HHLA2 expression and oncogenic pathway activity. Functionally, attenuation of MAPK/ERK signaling following HHLA2 depletion was accompanied by suppression of cell cycle progression and reduced clonogenic growth. Furthermore, alterations in epithelial–mesenchymal transition (EMT) markers were observed, including increased epithelial markers and decreased mesenchymal markers, indicating that HHLA2 may contribute to invasive and metastatic phenotypes (23, 24). These results collectively position HHLA2 as a potential modulator of tumor cell plasticity and aggressiveness. Importantly, these tumor-intrinsic effects appear to be at least partially independent of immune cell interactions, as they were observed *in vitro* in isolated cancer cell systems. This raises the possibility that HHLA2 may function beyond a classical immune ligand, potentially modulating tumor-intrinsic oncogenic signaling cascades. The mechanistic basis for this regulation remains to be fully elucidated, but several hypotheses can be proposed. First, HHLA2 may contribute to EGFR signaling stability at the plasma membrane. As a transmembrane protein, HHLA2 may influence receptor clustering, membrane microdomain organization, or lipid raft localization, thereby facilitating sustained EGFR activation. Second, HHLA2 might interact, either directly or indirectly, with adaptor proteins or scaffolding complexes that regulate downstream kinase phosphorylation dynamics. Finally, HHLA2 may potentially transmit intracellular reverse signals upon engagement with its receptors. Similar to other B7 family members, such reverse signaling could modulate intracellular kinase cascades within tumor cells and thereby contribute to HHLA2-mediated oncogenic amplification.

Taken together, current data support a model in which HHLA2 integrates tumor-intrinsic and immune-regulatory functions. By enhancing EGFR/MAPK/ERK pathway activation, HHLA2 may promote proliferation, invasion, and EMT, thereby coupling oncogenic signaling with immune checkpoint biology. This dual functionality underscores the complexity of HHLA2 in lung cancer and suggests that targeting HHLA2 may simultaneously disrupt tumor cell growth and immune evasion mechanisms.

## 4 HHLA2 and macrophage polarization: the tumor-associated macrophage axis

Beyond its tumor-intrinsic oncogenic functions, HHLA2 appears to actively shape the tumor microenvironment (TME), particularly through modulation of tumor-associated macrophages (TAMs) (25). TAMs represent one of the most abundant immune cell populations in lung cancer and play a pivotal role in regulating tumor progression, angiogenesis, immune suppression, and therapeutic resistance (26–28). Macrophages can broadly polarize toward a pro-inflammatory M1 phenotype or an immunosuppressive M2 phenotype, with the latter being associated with tumor-promoting functions (29–31). Emerging evidence suggests that HHLA2 contributes to macrophage reprogramming in NSCLC. The 2021 study in Cancer Medicine provided mechanistic insight into the role of HHLA2 in TAM polarization (13). Using NSCLC cell lines with stable HHLA2 knockdown, the authors demonstrated that depletion of HHLA2 significantly reduced the production and secretion of interleukin-10 (IL-10), a key anti-inflammatory cytokine known to drive M2 macrophage polarization. When conditioned media from HHLA2-deficient tumor cells were applied to THP-1-derived macrophages, a marked reduction in M2-associated markers—including CD163, CD206, Arg-1, and CCL18—was observed. In parallel, HHLA2 depletion was associated with increased expression of M1-associated markers, such as CD80 and inducible nitric oxide synthase (iNOS), suggesting that HHLA2 may not only promote M2 polarization but also suppress pro-inflammatory M1-like macrophage features. Flow cytometric analysis further confirmed a decreased proportion of CD163^+^ macrophages, indicating impaired M2 polarization.

These findings suggest that HHLA2 expression in tumor cells indirectly promotes an immunosuppressive macrophage phenotype through soluble mediators, particularly IL-10 (32, 33). Importantly, pharmacological inhibition of the MAPK/ERK pathway did not fully recapitulate the effects on IL-10 production, indicating that HHLA2-mediated cytokine regulation may occur through signaling mechanisms that are at least partially independent of canonical EGFR/MAPK activation. This observation reinforces the notion that HHLA2 participates in multiple parallel regulatory circuits within tumor cells. Functionally, M2-polarized TAMs contribute to tumor immune escape by secreting immunosuppressive cytokines (e.g., IL-10, TGF-β), inhibiting cytotoxic T-cell activity, promoting regulatory T-cell expansion, and facilitating extracellular matrix remodeling and angiogenesis. By driving IL-10 production and enhancing M2 polarization, HHLA2 may therefore create a permissive immune microenvironment that supports tumor growth and dampens anti-tumor immunity. These results expand the conceptual framework of HHLA2 biology. Rather than acting solely as a T-cell inhibitory ligand at the immune synapse, HHLA2 appears to function as a regulator of tumor–immune crosstalk. Through modulation of secreted cytokines, HHLA2 may orchestrate a broader immunosuppressive network that includes macrophages, T cells, and potentially other stromal components. This paracrine axis highlights HHLA2 as a central node linking tumor-intrinsic signaling to immune microenvironment remodeling.

**Figure 1:**
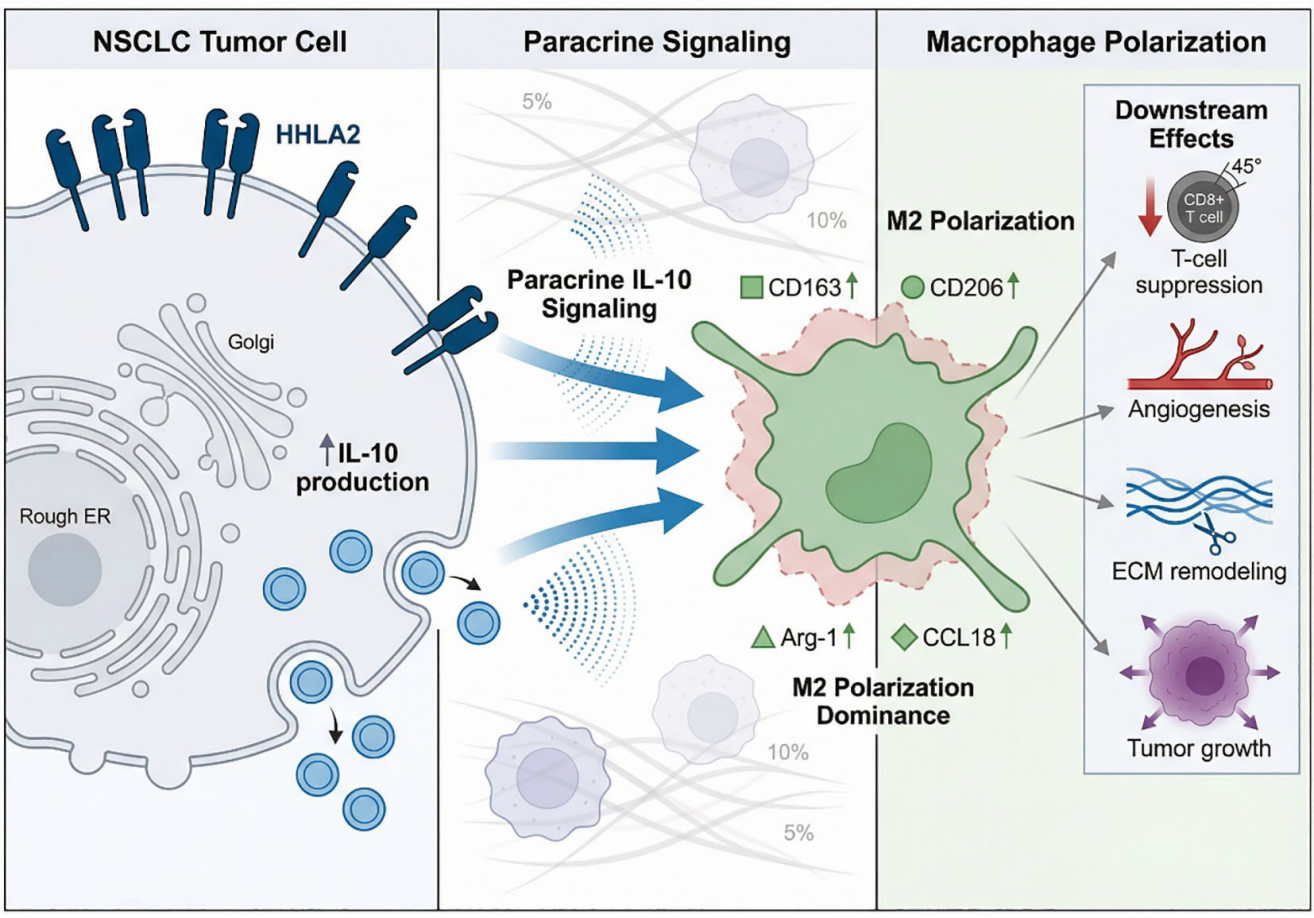
HHLA2 promotes IL-10–dependent M2 macrophage polarization in lung cancer. HHLA2 expressed on tumor cells enhances IL-10 secretion, which acts in a paracrine manner to promote M2 macrophage polarization. This leads to increased expression of M2 markers (CD163, CD206, Arg-1, CCL18) and contributes to an immunosuppressive tumor microenvironment, facilitating tumor progression and immune evasion.

Taken together, current evidence supports a model in which HHLA2 promotes lung cancer progression not only by enhancing oncogenic signaling within tumor cells but also by reshaping the TME toward an immunosuppressive state via IL-10–dependent M2 macrophage polarization. Targeting HHLA2 may therefore disrupt both tumor cell proliferation and macrophage-mediated immune suppression, providing a dual therapeutic advantage. **Figure 1** illustrates the role of HHLA2 in shaping the tumor microenvironment through IL-10–dependent macrophage polarization. HHLA2-driven M2 macrophage polarization contributes to immune suppression and tumor progression in lung cancer.

## 5 HHLA2–KIR3DL3 axis and CD8^+^ T-cell metabolic suppression

Among the emerging functions of HHLA2 in lung cancer, its role in directly suppressing cytotoxic T-cell metabolism represents a major conceptual advance. Recent work published in Cancer Letters (2026) identified the HHLA2–KIR3DL3 axis as a critical immune suppressive pathway in EGFR-mutant lung cancer, linking immune checkpoint signaling to metabolic regulation of CD8^+^ T cells (15). This finding not only clarifies the inhibitory receptor involved but also introduces a metabolic dimension to HHLA2-mediated immune evasion.

### 5.1 KIR3DL3 as an inhibitory receptor

KIR3DL3 is an inhibitory receptor expressed on subsets of cytotoxic T cells and natural killer (NK) cells (25, 34). Because KIR3DL3 is also expressed on NK cells, blockade of the HHLA2–KIR3DL3 interaction may restore NK cell-mediated tumor lysis, thereby extending the functional relevance of this axis beyond CD8^+^ T cells (35, 36). Unlike classical PD-1–mediated signaling, which primarily attenuates T-cell receptor (TCR) signaling and effector cytokine production, the HHLA2–KIR3DL3 interaction appears to exert broader functional effects. Engagement of KIR3DL3 by tumor-expressed HHLA2 was shown to suppress CD8^+^ T-cell activation, proliferation, and cytotoxicity in EGFR-mutant lung cancer models. Blocking this interaction restored T-cell effector function, suggesting that HHLA2 operates as a dominant inhibitory ligand in this molecular context. Importantly, HHLA2 expression was enriched in EGFR-mutant tumors, reinforcing the notion of a genotype-associated immune checkpoint. This association suggests that oncogenic EGFR signaling may contribute to upregulation of HHLA2, thereby establishing a tumor-intrinsic mechanism that promotes immune escape. In this model, EGFR-mutant tumor cells exploit HHLA2 expression to directly dampen cytotoxic T-cell responses via KIR3DL3 engagement.

### 5.2 Suppression of CD8^+^ T-cell glutamine metabolism

A particularly novel aspect of the HHLA2–KIR3DL3 axis is its impact on T-cell metabolism. The 2026 study demonstrated that engagement of KIR3DL3 by HHLA2 significantly reduced glutamine utilization in CD8^+^ T cells. Glutamine is a critical metabolic substrate that supports T-cell proliferation, nucleotide biosynthesis, redox balance, and effector molecule production (37–40). Limitation of glutamine metabolism leads to impaired cytokine secretion, diminished granzyme and perforin expression, and reduced cytolytic capacity (41, 42). Mechanistically, HHLA2–KIR3DL3 signaling was associated with downregulation of key metabolic enzymes and transporters involved in glutaminolysis, as well as suppression of metabolic signaling pathways that sustain anabolic processes in activated T cells. This metabolic restriction effectively places CD8^+^ T cells in a functionally exhausted or metabolically constrained state, even in the presence of antigen stimulation. The metabolic dimension of HHLA2-mediated suppression is particularly significant in the tumor microenvironment, where nutrient competition is already intense. Tumor cells often exhibit high glutamine consumption to support rapid proliferation and anabolic growth. By further limiting glutamine availability or utilization within T cells, HHLA2 amplifies the competitive disadvantage faced by anti-tumor immune cells. Thus, HHLA2 does not merely inhibit T-cell receptor signaling; it enforces a metabolic checkpoint that constrains immune effector function at a fundamental bioenergetic level.

### 5.3 Therapeutic synergy with EGFR tyrosine kinase inhibitors

The clinical relevance of this pathway is underscored by evidence that blockade of the HHLA2–KIR3DL3 axis enhances anti-tumor immunity and synergizes with EGFR tyrosine kinase inhibitors (TKIs). In EGFR-mutant lung cancer models, combined inhibition resulted in more robust tumor growth suppression compared with either strategy alone (43, 44). This synergy may reflect dual targeting of tumor-intrinsic oncogenic signaling and tumor-induced immune suppression. EGFR-TKIs reduce oncogenic signaling and tumor proliferation but often fail to generate durable immune-mediated control. Concurrent inhibition of HHLA2–KIR3DL3 may restore CD8^+^ T-cell metabolic competence and cytotoxicity, converting EGFR-targeted therapy into a more immunologically effective treatment. This combinatorial approach aligns with precision oncology principles, particularly for patients with EGFR-mutant NSCLC who historically derive limited benefit from conventional immune checkpoint blockade.

**Figure 2:**
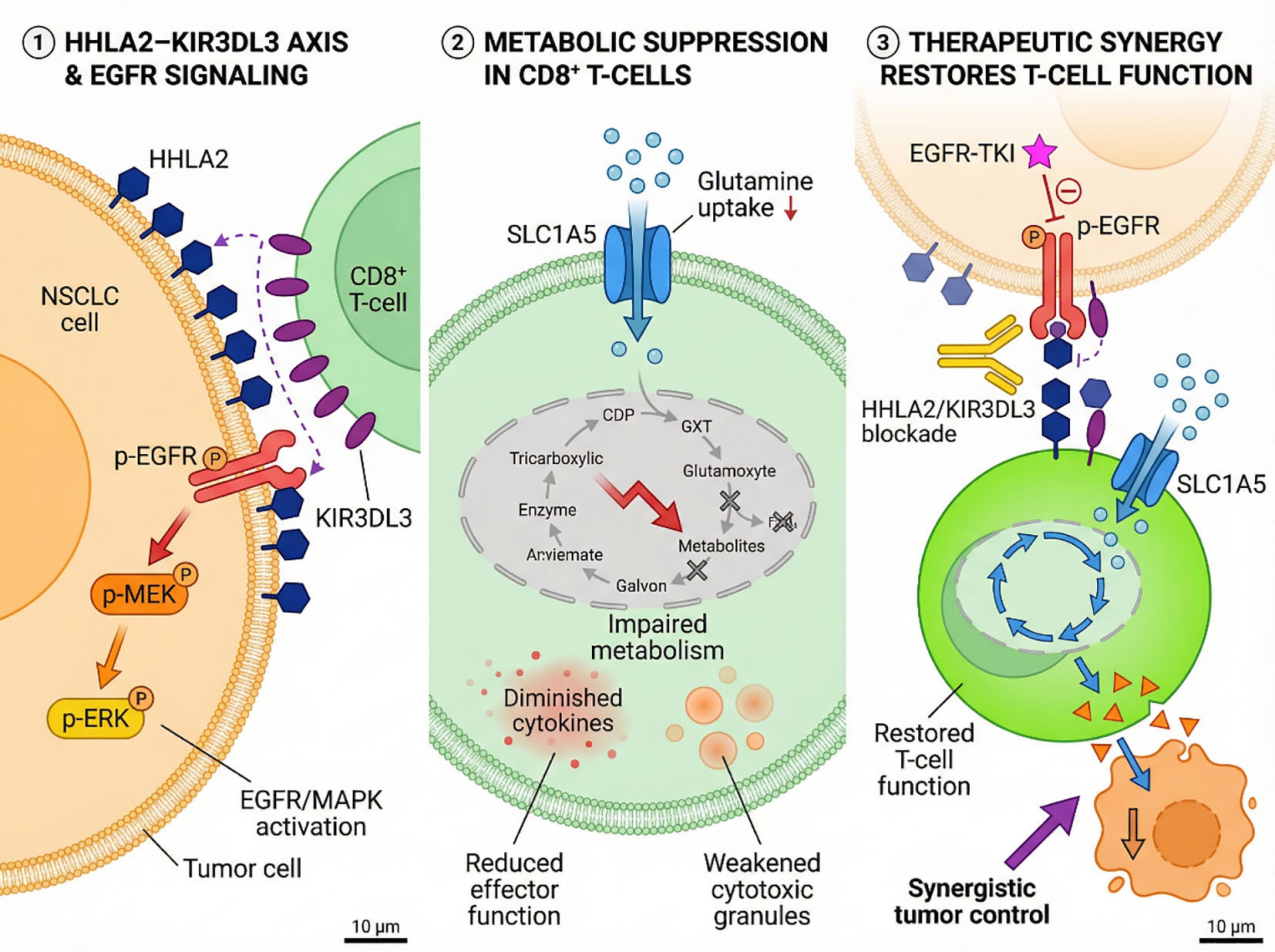
HHLA2–KIR3DL3 axis and CD8^+^ T-cell metabolic suppression in EGFR-mutant lung cancer. HHLA2 expressed on tumor cells binds to KIR3DL3 on CD8^+^ T-cells, inhibiting T-cell activation and glutamine metabolism. This metabolic restriction leads to impaired T-cell function and immune evasion. Targeting HHLA2–KIR3DL3 enhances T-cell activity and synergizes with EGFR tyrosine kinase inhibitors (TKIs) to suppress tumor growth.

Collectively, these findings support the concept that HHLA2 functions as a metabolic immune checkpoint. By integrating oncogenic EGFR signaling with suppression of T-cell glutamine metabolism, HHLA2 creates a coordinated system that promotes immune evasion in lung cancer. Targeting this axis may therefore disrupt both tumor cell growth and nutrient-based immune suppression, offering a promising avenue for genotype-guided immunotherapeutic strategies. **Figure 2** illustrates the HHLA2–KIR3DL3 axis in tumor-induced immune suppression, where HHLA2 engagement with KIR3DL3 reduces T-cell glutamine metabolism and cytotoxicity. Targeting this axis, in combination with EGFR-TKIs, restores T-cell function and enhances anti-tumor immunity in EGFR-mutant lung cancer.

## 6 Clinical implications and immunotherapy response

The biological relevance of HHLA2 in lung cancer extends beyond mechanistic insights into tangible clinical implications, particularly in the setting of immunotherapy. A recent study published in BMC Cancer (2024) provided important translational evidence linking HHLA2 expression to response to neoadjuvant immune checkpoint blockade in patients with NSCLC, especially those with chronic obstructive pulmonary disease (COPD) (45). This work highlights the potential utility of HHLA2 as a predictive biomarker and further supports its role in shaping the tumor immune microenvironment.

In a retrospective cohort of NSCLC patients receiving neoadjuvant immunotherapy followed by surgical resection, individuals with concomitant COPD exhibited significantly higher rates of major pathologic response (MPR) compared with non-COPD patients. Transcriptomic analyses and immunohistochemical validation revealed that HHLA2 expression was markedly reduced in tumors from COPD patients. Moreover, lower HHLA2 levels were associated with increased infiltration of CD8^+^CD103^+^ tissue-resident memory T (TRM) cells, a subset of T cells increasingly recognized as critical mediators of durable anti-tumor immunity. TRM cells reside within tumor tissues and provide rapid, localized immune responses upon antigen re-encounter. Their presence has been correlated with improved survival and enhanced responsiveness to immune checkpoint inhibitors (ICIs) across multiple cancer types (46–48). The observed inverse correlation between HHLA2 expression and TRM abundance suggests that HHLA2 may negatively regulate TRM maintenance or recruitment within the tumor microenvironment. Although the precise mechanisms remain to be clarified, it is plausible that HHLA2-mediated inhibition of T-cell activation or metabolic competence limits the establishment and persistence of functional TRM populations.

Importantly, patients whose tumors exhibited lower HHLA2 expression demonstrated improved pathologic responses following neoadjuvant immunotherapy. This finding implies that HHLA2 may contribute to primary resistance to ICIs and that reduced HHLA2 expression creates a more permissive immune environment for effective anti-tumor responses. Given that EGFR-mutant tumors tend to express higher levels of HHLA2 and are often less responsive to ICIs, these data further reinforce the concept of HHLA2 as a genotype-associated immune suppressive factor with predictive significance. Clinically, these observations position HHLA2 as a potential biomarker for patient stratification in immunotherapy. Assessing HHLA2 expression levels may help identify individuals more likely to benefit from ICI-based regimens or, conversely, those who may require combinatorial strategies targeting the HHLA2–KIR3DL3 axis. Furthermore, modulation of HHLA2 expression could potentially enhance TRM stability and improve therapeutic outcomes.

Overall, emerging clinical evidence suggests that HHLA2 not only participates in immune suppression at the molecular level but also influences treatment responsiveness in real-world patient cohorts. Continued investigation into HHLA2-guided therapeutic strategies may help refine precision immunotherapy approaches in lung cancer.

## 7 Challenges and future perspectives

Despite growing evidence supporting HHLA2 as a clinically relevant immune checkpoint in lung cancer, several important challenges remain to be addressed. One major limitation lies in its evolutionary restriction. HHLA2 is a primate-specific molecule and is absent in standard murine models, which significantly constrains *in vivo* mechanistic studies and preclinical therapeutic testing (11, 49). While humanized mouse models and ex vivo systems partially overcome this barrier, they do not fully recapitulate the complexity of tumor–immune interactions in patients. The development of more physiologically relevant experimental platforms will be essential to accurately evaluate HHLA2-targeted strategies. Another unresolved issue concerns receptor specificity and context-dependent signaling. HHLA2 can bind to both KIR3DL3, an inhibitory receptor, and TMIGD2 (CD28H), which has been described as a co-stimulatory receptor expressed on naïve T cells and other immune subsets. The relative expression patterns of these receptors in tumor-infiltrating lymphocytes remain incompletely characterized, particularly across disease stages and treatment contexts. It is possible that HHLA2 exerts divergent effects depending on receptor dominance within the tumor microenvironment. Clarifying receptor distribution, signaling hierarchy, and cell-type specificity will be critical for therapeutic targeting.

The relationship between HHLA2 and the PD-1/PD-L1 axis also warrants further investigation. Whether HHLA2 functions redundantly, synergistically, or independently of PD-1 signaling remains unclear. Given that EGFR-mutant tumors often exhibit suboptimal responses to PD-1 blockade, HHLA2 may represent a complementary or compensatory immune suppressive pathway. Comprehensive co-expression and combinatorial blockade studies are needed to define whether dual targeting of HHLA2 and PD-1/PD-L1 could overcome primary resistance. From a therapeutic perspective, HHLA2 presents multiple translational opportunities. Monoclonal antibodies targeting HHLA2 or KIR3DL3 could restore cytotoxic T-cell function. Bispecific antibodies engaging HHLA2 alongside other immune checkpoints may enhance efficacy. Additionally, HHLA2-directed CAR-T or CAR-NK approaches could potentially exploit its tumor-associated expression pattern. In particular, the possibility that HHLA2–KIR3DL3 blockade may restore NK cell-mediated tumor lysis provides a mechanistic rationale for integrating HHLA2 targeting with CAR-NK–based strategies. Finally, given its enrichment in EGFR-mutant tumors, HHLA2 may serve as a stratification biomarker for genotype-guided combination therapy with EGFR tyrosine kinase inhibitors.

In summary, although significant challenges remain, HHLA2 represents a promising immunotherapeutic target at the intersection of oncogenic signaling, metabolic regulation, and immune suppression. Continued mechanistic and translational research will determine whether targeting this pathway can meaningfully improve outcomes for patients with lung cancer.

## 8 Conclusion

HHLA2 has emerged as a multifaceted immune checkpoint molecule that occupies a unique position at the intersection of oncogenic signaling and immune regulation in lung cancer. Accumulating evidence indicates that HHLA2 is widely expressed in both NSCLC and SCLC, with particularly strong enrichment in EGFR-mutant tumors. Beyond its canonical role as a B7 family ligand modulating T-cell activity, HHLA2 appears to exert tumor-intrinsic effects by enhancing EGFR/MAPK/ERK signaling, promoting proliferation and epithelial–mesenchymal transition, and facilitating tumor progression. Simultaneously, HHLA2 contributes to immune microenvironment remodeling through IL-10–dependent macrophage polarization and suppression of CD8^+^ T-cell glutamine metabolism via KIR3DL3 engagement. This dual functionality underscores a broader conceptual shift: HHLA2 should not be viewed solely as a surface immune inhibitory molecule, but rather as a genotype-associated immune checkpoint that integrates tumor cell–intrinsic oncogenic programs with metabolic and cellular mechanisms of immune escape. Its association with reduced tissue-resident memory T-cell infiltration and diminished responsiveness to immunotherapy further supports its clinical relevance. Importantly, emerging data suggest that targeting the HHLA2–KIR3DL3 axis may synergize with EGFR tyrosine kinase inhibitors, offering a rational precision strategy for EGFR-mutant lung cancer—a subgroup that has historically derived limited benefit from conventional immune checkpoint blockade. While significant mechanistic and translational questions remain, current findings collectively position HHLA2 as a promising biomarker and therapeutic target. Continued investigation into its receptor interactions, metabolic effects, and combinatorial treatment potential may pave the way for more effective, genotype-guided immunotherapy approaches in lung cancer. 
